# Physicochemical and Biochemical Evaluation of Amorphous Solid Dispersion of Naringenin Prepared Using Hot-Melt Extrusion

**DOI:** 10.3389/fnut.2022.850103

**Published:** 2022-04-27

**Authors:** Kenji Ishimoto, Yukiko Shimada, Akane Ohno, Shuichi Otani, Yukio Ago, Soya Maeda, Bangzhong Lin, Kazuto Nunomura, Nobumasa Hino, Masayuki Suzuki, Shinsaku Nakagawa

**Affiliations:** ^1^Laboratory of Biopharmaceutics, Graduate School of Pharmaceutical Sciences, Osaka University, Osaka, Japan; ^2^Laboratory of Innovative Food Science, Graduate School of Pharmaceutical Sciences, Osaka University, Osaka, Japan; ^3^Global Center for Medical Engineering and Informatics, Osaka University, Osaka, Japan; ^4^Center for Supporting Drug Discovery and Life Science Research, Graduate School of Pharmaceutical Sciences, Osaka University, Osaka, Japan; ^5^Mitsui Norin Co., Ltd., R&D Group, Shizuoka, Japan; ^6^Department of Cellular and Molecular Pharmacology, Graduate School of Biomedical and Health Sciences, Hiroshima University, Hiroshima, Japan

**Keywords:** amorphous solid dispersion, analgesia, functional food, gastrointestinal absorption, hot-melt extrusion, naringenin, poor water solubility, toxicity

## Abstract

Naringenin (NRG) is a plant-derived flavonoid. Due to its antioxidant, anti-inflammatory, and analgesic activities it is beneficial to human health and is often used as a functional food ingredient; however, it has poor water solubility and low *in vivo* bioavailability. Therefore, the efficacy of NRG can be improved by enhancing its water solubility to increase gastrointestinal absorption. Conventional methods for the formulation of NRG are very complex and use toxic organic solvents, making them impractical for the production of functional foods. The objective of this study was to develop a safe and effective NRG-based functional food material. Previously, we established a technology to prepare amorphous solid dispersions (SDs) from functional food ingredients with poor water solubility and used hot-melt extrusion technology that is comparatively simple and does not involve the use of organic solvents. In this study, we prepared NRG SD and evaluated them both physicochemically and biochemically. NRG SD had superior water solubility and gastrointestinal absorption relative to native NRG and showed higher analgesic efficacy in rats than crystalline NRG. NRG SD was administered to mice in a mixed diet for 28 days, and organ weights and hematological/clinical biochemical parameters were assessed. NRG SD did not demonstrate severe adverse effects. The results suggest that NRG SD is a safe and highly efficacious formulation that can be used as a functional food material in the future.

## Introduction

In addition to a healthy lifestyle, the consumption of over-the-counter health foods, dietary supplements, medicinal plants, and other natural resources can help maintain good health and reduce the risk of chronic diseases (1). Although the sale of products containing functional ingredients has been steadily increasing, the field of functional medicine faces important challenges such as inadequate knowledge of the mechanisms, side effects, and contraindications of these ingredients or their interactions with drugs and other functional ingredients (2). Moreover, certain functional food ingredients have poor water solubility. Hence, their absorption in the gastrointestinal tract and bioavailability are limited (3). For these reasons, the safety, quality, and efficacy of functional food ingredients should be determined prior to their application.

Naringenin (NRG) is a functional food ingredient derived from grapefruit, orange, and tomato (4). This flavonoid compound shows antioxidant, anti-inflammatory, anti-obesity, and analgesic activities (5–8). However, it has poor water solubility and low *in vivo* bioavailability (9). Therefore, an appropriate formulation for improved water solubility and gastrointestinal absorption is required. Various methods have been implemented to enhance the water solubility of NRG, including the use of nanoparticles, β-cyclodextrin inclusion complexes, self-nanoemulsifying drug delivery systems, and submicron carriers (10–13). Shulman et al. reported improved gastrointestinal NRG absorption in rats fed a soluble complex consisting of NRG and hydroxypropyl-β-cyclodextrin (14). However, these methods are not suitable for preparing NRG as a functional food because they are highly complex and involve contamination of the final product with organic solvents. Therefore, novel NRG-based functional foods should be developed to overcome these problems and produce safe, effective, and economical functional foods that reflect the physiological effects of NRG that are beneficial to human health.

To resolve these challenges and to enable functional foods to retain beneficial properties for human health, we have basically investigated methods to prepare amorphous solid dispersions (SDs) of functional food ingredients with poor water solubility (15). The SD technique is a promising strategy to increase the water solubility of hydrophobic molecules by dispersing them in carriers such as polymers and sugar alcohols (3, 16). Our SDs are prepared by mixing food-grade polymers and emulsifiers with low-solubility food ingredients at high temperature. Previous studies showed that amorphization of a functional food ingredient with poor water solubility, such as β-carotene, dramatically improved its gastrointestinal absorption (15, 17, 18). The main advantage of this method is that it develops safe formulations, and all functional and base materials are food-grade substances. Moreover, no organic solvents are used in the process, and no potentially toxic residues are produced. This manufacturing process also supports large-scale production (19). Although the pharmaceutical industry has applied hot-melt extrusion to manufacture a variety of commercial products available in the market, this method has rarely been used for the commercialization of functional food ingredients (20). Recent studies also reported improvement of the solubility of functional food ingredients such as cocoa extract, *Ginkgo biloba*, and curcumin using the hot-melt extrusion technology (21–23). Granados et al. used hot-melt extrusion technology to prepare complexes of NRG, hydrophilic polymers, and cyclodextrin (24). However, the pharmacokinetics, functionality, and safety of these NRG complexes have not been evaluated, and thus, such complexes have not been used widely in practical applications. Nevertheless, previous research has contributed to the increasing attention on the application and practical use of functional food materials prepared by hot-melt extrusion technology.

We hypothesized that the proposed method may aid in the preparation of NRG SD and formulation of safe food substances and retain a high therapeutic efficacy of NRG. Our prior research demonstrated that this method was effective only for β-carotene, which has a carotenoid skeleton consisting only of carbon and hydrogen. It remains unknown whether an effective amorphous SD can be produced from NRG with a flavonoid skeleton. In the present study, we aimed to examine the physicochemical properties, gastrointestinal absorption, efficacy, and safety of NRG SD prepared using the hot-melt extrusion technology and investigated the potential of this method to generate a safe and efficacious NRG-based functional food material.

## Materials and Methods

### Materials

Naringenin (NRG, >98.0%) and mefenamic acid were purchased from Tokyo Chemical Industry Co., Ltd. (Tokyo, Japan). Polyvinylpyrrolidone (PVP) Kollidon 25 was provided by BASF Japan Ltd. (Tokyo, Japan). Polyglycerol fatty acid ester O-50D (PG) was obtained from Mitsubishi Chemical Foods Co., Ltd. (Tokyo, Japan). Dissolution test solutions 1 and 2 were purchased from Nacalai Tesque, Inc. (Kyoto, Japan). β-Glucuronidase from *Helix pomatia* Type HP-2 (No. G7017; β-glucuronidase activity ≥ 100,000 U/mL; sulfatase activity = 7,500 U/mL) and daidzein were purchased from Sigma-Aldrich Corp. (St. Louis, MO, United States). FUJI DRI-CHEM slides for clinical biochemistry analysis were purchased from FUJIFILM Corporation (Tokyo, Japan).

### Animals

All animal study protocols were approved by the Animal Care and Use Committee of the Graduate School of Pharmaceutical Sciences Osaka University, Osaka, Japan. All experimental procedures were conducted in accordance with the Guide for the Care and Use of Laboratory Animals (25). Extra care was taken to minimize animal suffering and the number of animals used. Male Wistar rats and male and female ICR mice were obtained from Japan SLC Inc. (Shizuoka, Japan) and acclimatized under controlled environmental conditions (22 ± 1°C; 50% ± 10 relative humidity; 12-h light-dark cycle; lights on at 8:00) for at least several days before the start of the experiment. All animals were fed a certified MF diet (Oriental Yeast Co., Ltd., Tokyo, Japan) and had *ad libitum* access to water.

### Preparation of Naringenin Solid Dispersion by Twin-Screw Extrusion

NRG, PVP, and PG were mixed in a 1:8.5:0.5 (w/w/w) ratio. To prepare the NRG SD, the physical mixtures (PMs) were heat-kneaded with a twin-screw extruder (Technovel Co., Ltd., Osaka, Japan; NARA Machinery Co., Ltd., Tokyo, Japan) (17). The extrusion temperature range was 180–200°C and the screw speed was 100 rpm. The product was cooled in the dark at 20–25°C, pulverized in an agate mortar, and passed through a 150-μm sieve. The SD was then placed in an amber bottle and stored in a desiccator at 20–25°C.

### Solubility Test and High-Performance Liquid Chromatography Analysis

The prepared NRG SD (500 mg; equivalent to 50 mg NRG) was added to 2.5 mL dissolution test solution 2. The mixture was shaken vigorously at 37°C for 30 min and centrifuged (15, 17). The supernatant was then passed through a 0.2-μm filter and subjected to high-performance liquid chromatography (HPLC) analysis. The NRG concentration was determined using an HPLC system fitted with the LC-20AD pump and the SPD-M20 detector (Shimadzu Corp., Kyoto, Japan) (15). A reverse-phase column (CAPCELL PAK C18 MGII; 4.6 mm × 100 mm; 5 μm; Shiseido Co., Ltd., Tokyo, Japan) was operated at 40°C. The mobile phase consisted of a methanol-water (45:55, v/v) mixture with 0.05% (v/v) phosphoric acid, and it was ultrasonically degassed before use. The flow rate was set at 1 mL/min, and the detection wavelength was set at 289 nm. The sample injection volume was 10 μL. The HPLC was calibrated using standard methanol-water (50:50, v/v) NRG solutions in the concentration range of 0.125–40 μg/mL.

### Naringenin Solid Dispersion Dissolution Test

The dissolution test was conducted using the paddle method described in the Japanese Pharmacopoeia (26). First, 900 mL dissolution test solution 1 or 2 was placed in the dissolution tester vessel and then 30 mg of crystalline NRG, 300 mg of PM, and 300 mg of SD were added to it. The temperature (37°C) and stirring rate (100 rpm) were maintained during the experiment. Samples were collected 2, 5, 10, 20, 30, 45, 60, 90, and 120 min after sample addition. The solutions were passed through 0.2-μm filters, and the filtrates were subjected to HPLC analysis.

### Evaluation of the Physical Properties of Naringenin Solid Dispersion by Differential Scanning Calorimetry

Sample thermograms were obtained using a differential scanning calorimeter (ThermoPlusEVOII DSC8230; Rigaku Corp., Tokyo, Japan). Samples were weighed to 5 mg, placed in covered aluminum pans, and heated from 25 to 275°C at 20°C/min in an atmosphere containing nitrogen.

### Evaluation of the Physical Properties of Naringenin Solid Dispersion by Powder X-Ray Diffraction

The crystalline state of the sample was analyzed *via* automated multipurpose X-ray diffractometry (SmartLab; Rigaku Corp.) fitted with a D/teX Ultrahigh-Speed 1D X-ray Detector (Rigaku Corp.). The X-ray source was CuKα radiation fitted with a CuKβ filter. The X-ray output was 45 kV and 200 mA. Samples were analyzed over a 5–50° diffraction angle range (2θ) at 10°/min.

### Pharmacokinetics of Naringenin Solid Dispersion After Oral Administration

Rats aged 9 weeks were fasted for 16 h and intragastrically administered 100 mg/kg body weight (BW) crystalline NRG, PM equivalent to 100 mg/kg BW NRG, or SD equivalent to 100 mg/kg BW NRG. Each solution was suspended in ion-exchange water immediately prior to administration. Blood was collected from the tail vein 0.125, 0.25, 0.5, 1, 2, 4, and 8 h after administration. Blood samples were also collected from the heart under isoflurane anesthesia 24 h after administration. The blood was stored in heparinized hematocrit tubes, and the plasma was isolated by centrifugation.

### Preparation of the Solution for Intravenous Administration

NRG SD equivalent to 200 mg/kg BW NRG was intragastrically administered to 9-week-old rats that were fasted for 16 h. The same amount of NRG SD was intragastrically administered 5 min later. Blood was collected from the heart under isoflurane anesthesia 35 min after the first administration and stored at 4°C for several hours. Serum was then isolated by centrifugation and analyzed by liquid chromatography-tandem mass spectrometry (LC-MS/MS). The serum contained 34.8 μg/mL unchanged NRG and 134.5 μg/mL NRG metabolites.

### Pharmacokinetics of Naringenin Solid Dispersion After Intravenous Administration

Serum containing the unchanged NRG and NRG metabolites was administered *via* the tail vein at a dose of 500 μL/rat. Blood was collected from the tail vein 2.5, 5, 7.5, 10, 12.5, 20, 40, and 80 min after administration and stored in heparinized hematocrit tubes. The plasma was isolated by centrifugation.

### Extraction of Naringenin From Plasma or Serum and Liquid Chromatography-Tandem Mass Spectrometry Analysis

To extract the unchanged NRG, 5 μL of plasma derived from rats that were orally or intravenously administered NRG SD was mixed with 20 μL of normal rat plasma, 5 μL of 90% (v/v) acetonitrile, and 50 μL of acetonitrile containing 2 μM daidzein and 0.1 μM mefenamic acid. The mixture was centrifuged at 4°C, and 25 μL supernatant was collected and mixed with 75 μL of 30% (v/v) acetonitrile.

To prepare NRG metabolites, 6 μL of plasma derived from rats that were orally or intravenously administered NRG SD was mixed with 3 μL of 0.2 M sodium acetate buffer (pH 5.0) containing 0.2% (w/v) ascorbic acid, 1.2% (w/v) EDTA, and 10 U/μL β-glucuronidase (Type HP-2 from Helix pomatia). The samples were incubated at 37°C overnight and extracted using the same method that was employed for those containing the unchanged NRG.

LC-MS/MS analysis was performed using an ACQUITY UPLC system (Waters Corp., Milford, MA, United States) fitted with a cooling autosampler and a column oven with temperature control. Mass spectrometry was performed using a Waters Xevo TQ-S tandem quadrupole mass spectrometer (QQ-MS; Waters Corp.). Chromatographic separation was performed on an ACQUITY UPLC BEH C18 1.7 μm column (50 mm × 2.1 mm; Waters Corp.). The mobile phase A was 0.1% formic acid in water (v/v), and acetonitrile containing 0.1% formic acid was used as mobile phase B. The flow rate was set at 0.5 mL/min. The sample injection volume was 5 μL. The injected sample was eluted from the column with the following gradient program: 1.8-min linear gradient in solvent B from 2 to 98%, 0.7-min linear gradient in solvent B from 98 to 2%, and 2.5-min hold at 2% B. The sample injection volume was 5 μL. Positive electrospray ionization was used to generate the parental ions. Multiple reaction monitoring, using precursor → product ion transitions of m/z 273.04 → 152.97 and m/z 255.10 → 198.99, was used to quantify NRG and daidzein (internal standard), respectively.

### Pharmacokinetic Analysis

Pharmacokinetic analyses after oral or intravenous NRG administration were performed using the free Napp software, which was provided by the University of Tokyo Hospital (27).

### Acetic Acid-Induced Writhing Test

The acetic acid-induced writhing test was performed according to the method of Pinho-Ribeiro et al. (8) with modifications. Rats aged 5–7 weeks were fasted for 16 h and divided into four groups of eight animals per group. The first group was intragastrically administered 10 mL/kg saline as a control. The second group was intragastrically administered crystalline NRG at 100 mg/kg BW. The third and fourth groups were intragastrically administered NRG SD equivalent to 30 mg/kg and 100 mg/kg BW NRG, respectively. After 5 min, each rat was intraperitoneally injected with 10 mL/kg of 0.8% (v/v) acetic acid in saline solution. Visceral pain intensity was quantified by enumerating the abdominal writhing within 0–25 min after the stimulus. The writhing response was characterized by the contraction of abdominal muscles and stretching of the hind limbs.

### Safety Test

Mice aged 6 weeks were randomly divided into two groups of 10 mice (five males and five females) per group. The experimental group was prepared by mixing NRG SD with powdered food to a concentration of 1% (w/w) NRG SD. The control group was prepared by mixing the base material (PVP:PG = 8.5:1 weight ratio) with powdered food to a concentration of 1% (w/w). After the mixed diet was administered, body weight, food, and water consumption were measured for each mouse group for 28 days. On day 28, all animals were anesthetized with chloral hydrate, and blood samples were collected from the heart and stored in heparinized tubes. Portions of these samples were utilized to evaluate hematological parameters using an XT-2000i hematology analyzer (Sysmex Corp., Hyogo, Japan). The remaining blood samples were centrifuged to obtain the plasma for which comprehensive clinical chemistry test parameters were evaluated using a DRI-CHEM 4000V veterinary chemistry analyzer (FUJIFILM Corp., Tokyo, Japan). The livers, spleens, hearts, kidneys, lungs, and brains were excised and weighed. The organ weights were divided by body weight measured on day 28.

### Statistical Analysis

All data are presented as mean ± standard error. Statistical analysis was performed with the Tukey-Kramer method or an unpaired *t*-test method using Statcel v.4.0 (OMS Publishing Inc., Saitama, Japan).

## Results

### Preparation of Naringenin Solid Dispersion by Twin-Screw Extrusion

**FIGURE 1:**
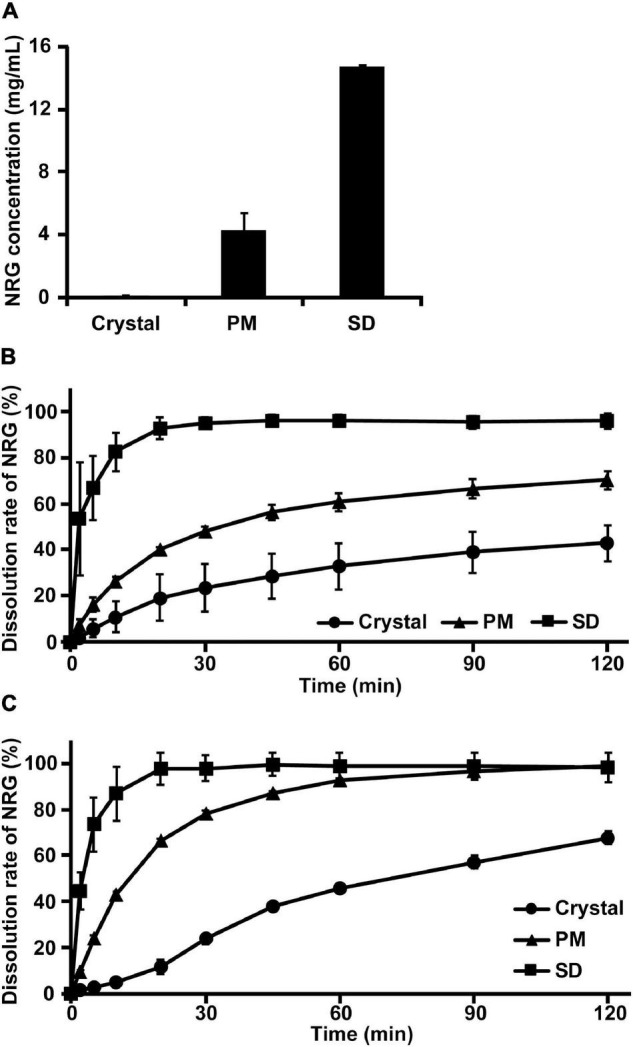
Water solubility and dissolution rate of amorphized naringenin (NRG) solid dispersions (SD). **(A)** NRG solubility was determined for crystalline NRG, PM, and NRG SD suspensions in Japanese Pharmacopoeia dissolution test solution 2 (pH 6.8) (mean ± SE]; *n* = 3). **(B)** NRG dissolution rates when crystalline NRG, PM, and NRG SD were added to Japanese Pharmacopoeia dissolution test solution 1 (pH 1.2) (mean ± SE; *n* = 3). **(C)** NRG dissolution rates when crystalline NRG, PM, and NRG SD were added to Japanese Pharmacopoeia dissolution test solution 2 (pH 6.8) (mean ± SE; *n* = 3). For **(B,C)** NRG SD was thermally mixed at 200°C.

Preliminary experiments were conducted on the base materials and their mixtures with various ratios *via* previously reported handmade hot-melt technology (15). The preparation of SD by heat-kneading the materials at a 1:8.5:0.5 (w/w/w) NRG:PVP:PG mixing ratio substantially increased the solubility of NRG in water (data not shown). We prepared NRG SD using a twin-screw extruder by feeding a 1:8.5:0.5 (w/w/w) NRG:PVP:PG mixture into the extruder and mixing it at high temperature (17). We then compared the water solubility of NRG SD, crystalline NRG, and PM. The latter was the product formed by mixing NRG, PVP, and PG at 1:8.5:0.5 (w/w/w). The water solubility values were 70 μg/mL, 4 mg/mL, and 15 mg/mL for crystalline NRG, PM, and SD, respectively (Figure 1A). Therefore, using the twin-screw extrusion method, the synthesized NRG SD had excellent solubility.

### Naringenin Solid Dispersion Dissolution Characteristics

The dissolution characteristics of the highly water-soluble NRG SD were evaluated using Japanese Pharmacopoeia-based dissolution tests (26). Dissolution test solutions 1 (pH 1.2) and 2 (pH 6.8) were used. Only 40 and 60% of the crystalline NRG was dissolved in test solutions 1 and 2, respectively, after 120 min (Figures 1B,C). Only 40 and 70% of crystalline NRG and PM were dissolved in test solution 1, respectively, after 120 min. By contrast, 100% of NRG SD was dissolved in test solution 1 after 30 min (Figure 1B). Approximately, 70% of crystalline NRG was dissolved in test solution 2 after 120 min, whereas 100% of the PM and SD were dissolved in test solution 2 after 90 min and 20 min, respectively (Figure 1C). Hence, NRG SD dissolved faster in test solutions 1 and 2 than either crystalline NRG or PM and hence, it is expected to be rapidly absorbed in the body.

### Physicochemical Properties of Naringenin Solid Dispersion

**FIGURE 2:**
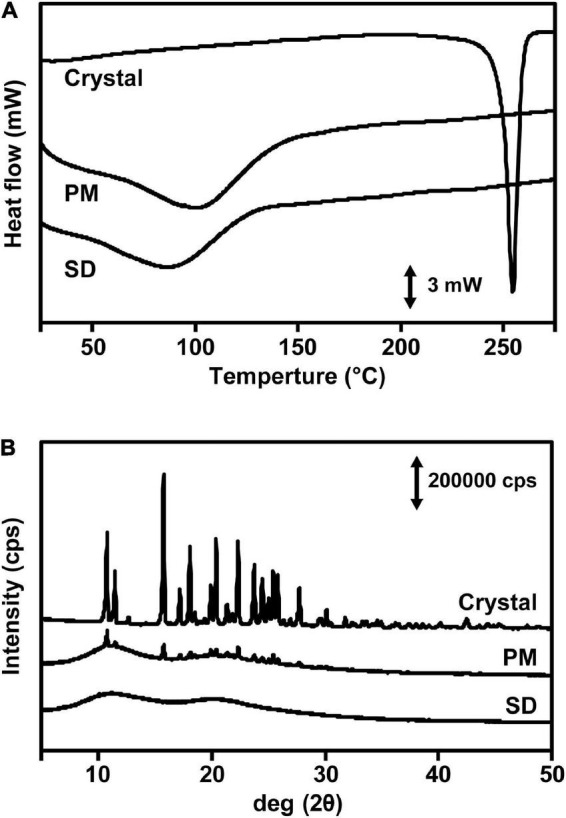
Physicochemical evaluation of NRG SD. **(A)** DSC thermograms of crystalline NRG, PM, and NRG SD. **(B)** PXRD analyses of crystalline states of NRG, PM, and NRG SD.

The water solubility of the prepared NRG SD was superior to those of crystalline NRG and PM. However, it was unknown whether the SD was, in fact, amorphous. We investigated the crystalline state of NRG SD *via* differential scanning calorimetry (DSC) and powder X-ray diffraction (PXRD). The DSC thermogram of crystalline NRG showed a sharp endothermic peak near 250°C corresponding to the NRG melting point (Figure 2A). However, this peak was absent in the PM and SD thermograms. The PXRD analysis revealed strong diffraction peaks between 10° and 30° for the NRG crystals, thereby confirming their crystalline form (Figure 2B). For PM, the characteristic crystalline NRG peaks remained intact and their intensity only slightly changed. In contrast, for SD, the crystalline NRG exhibited a diffuse broad peak and lacked the intensity of the peaks observed for PM. For these reasons, NRG underwent a phase transition from the crystalline to the amorphous form during preparation of SD.

### Changes in Plasma Naringenin Concentration After Administration of Oral Naringenin Solid Dispersion

**FIGURE 3:**
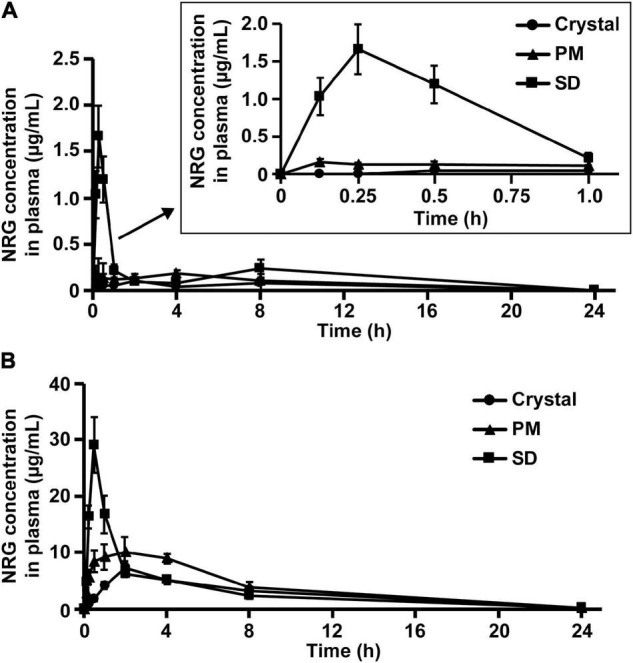
NRG levels in rat plasma after oral administration of NRG SD. Plasma concentrations of unchanged NRG **(A)** and NRG metabolites **(B)** after oral administration of crystalline NRG, PM, and NRG SD. NRG equivalent to 100 mg/kg was administered to rats (means ± SE; *n* = 6).

To investigate gastrointestinal NRG SD absorption, we measured the plasma levels of crystalline NRG, PM, and SD that were orally administered to rats. The area under the plasma concentration-time curve (AUC_0–∞_) was 0.74 ± 0.24 μg⋅h/mL, the maximum plasma concentration (C_max_) was 0.13 ± 0.01 μg/mL, and the time required to reach maximum plasma concentration (t_max_) was 4.00 ± 1.26 h in the rats administered 100 mg/kg crystalline NRG (Figure 3A and Table 1). The results for the rats that were administered with SD at a dose equivalent to 100 mg/kg NRG were AUC_0–∞_ = 1.99 ± 0.46 μg⋅h/mL, C_max_ = 1.67 ± 0.33 μg/mL, and t_max_ = 0.29 ± 0.04 h. The rats administered SD showed a 2.7-fold increase in AUC_0–∞_, a 12.8-fold increase in C_max_, and a 3.7 h decrease in t_max_ compared to those administered crystalline NRG. A 1.5-fold increase in AUC, a 1.8-fold increase in C_max_, and a 2.4 h decrease in t_max_ were observed in rats administered PM at a dose equivalent to 100 mg/kg NRG. In terms of AUC_0–1 h_, there was a 3.7-fold increase in PM and a 26.4-fold increase in SD relative to crystalline NRG. The amorphization of unchanged NRG enabled it to be rapidly absorbed in the gut. No significant differences in AUC_0–1 h_ were observed among crystalline NRG, PM, and SD in terms of plasma NRG glucuronide and sulfate conjugates. However, SD showed a 3.9-fold increase in C_max_ and a 1.9 h decrease in t_max_ compared to crystalline NRG (Figure 3B and Table 1). NRG SD demonstrated substantially improved gastrointestinal absorption, especially at a very high plasma NRG concentration immediately after oral administration.

**TABLE 1:** Pharmacokinetic profiles of crystalline NRG, PM, and NRG SD orally administered to rats.

Pharmacokinetic parameter	Unchanged NRG			NRG metabolites		
	NRG	PM	ASD	NRG	PM	ASD
AUC_0–∞_ (μg⋅h/mL)	0.74 ± 0.24	1.08 ± 0.23	1.99 ± 0.46	51.68 ± 9.83	70.45 ± 10.15	61.84 ± 8.46
AUC_0–1 h_ (μg⋅h/mL)	0.03 ± 0.01	0.12 ± 0.03	0.86 ± 0.15	1.76 ± 0.29	6.80 ± 1.55	18.20 ± 2.42
AUC_0–t_ (μg⋅h/mL)	0.74 ± 0.24	1.08 ± 0.23	1.99 ± 0.46	51.60 ± 9.81	70.42 ± 10.15	61.81 ± 8.46
C_max_ (μg/mL)	0.13 ± 0.01	0.24 ± 0.04	1.67 ± 0.33	7.46 ± 1.23	11.63 ± 2.07	29.02 ± 4.92
t_max_ (μg/mL)	4.00 ± 1.26	1.65 ± 0.75	0.29 ± 0.04	2.50 ± 0.50	2.42 ± 0.55	0.58 ± 0.08
F (%)	0.52 ± 0.17	0.75 ± 0.15	1.43 ± 0.33	N/A	N/A	N/A

*AUC, area under the plasma concentration-time curve; C_max_, maximum plasma concentration; t_max_, time of maximum plasma concentration; F, absolute bioavailability; N/A, not applicable.*

### Pharmacokinetic Profile of Orally Administered Naringenin Solid Dispersion in Rats

**FIGURE 4:**
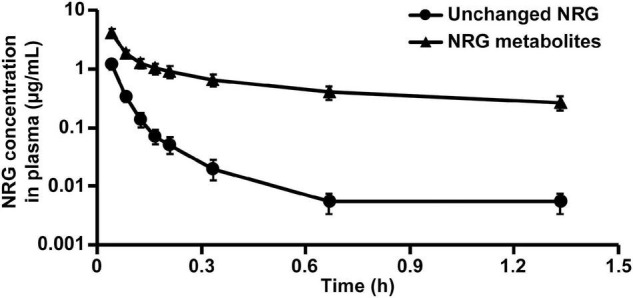
Rat plasma NRG concentrations after intravenous administration of unchanged NRG and NRG metabolites. Serum containing 34.8 μg/mL unchanged NRG or 134.5 μg/mL NRG metabolites was administered to rats at 500 μL (means ± SE, *n* = 4).

We evaluated the pharmacokinetic parameters of NRG to characterize NRG SD. We prepared sera containing unchanged NRG and NRG metabolites, intravenously administered them to the rats, and measured their unchanged NRG and NRG metabolite levels in the plasma. The transitions in the concentrations of unchanged NRG and NRG metabolites in the plasma were biphasic (Figure 4). Hence, their pharmacokinetic parameters were evaluated using a two-compartment model (Table 2). The absolute bioavailability values (F) of crystalline NRG, PM, and SD were 0.52, 0.75, and 1.43%, respectively. Therefore, the amorphization of NRG *via* hot-melt extrusion technology increased its F by ∼2.7-fold. These results suggest that NRG SD may strongly exhibit the physiological functions of NRG.

**TABLE 2:** Pharmacokinetic profiles of intravenously administered unchanged NRG and NRG metabolites in rats.

Pharmacokinetic parameter	Unchanged NRG	NRG metabolites
AUC_0–∞_ (μg⋅h/mL)	0.13 ± 0.02	1.35 ± 0.28
AUC_0–*t*_ (μg⋅h/mL)	0.12 ± 0.02	1.08 ± 0.22
CL (L/h)	0.16 ± 0.04	0.06 ± 0.01
Vd (L/kg)	0.19 ± 0.08	0.18 ± 0.04
t_1/2_ (h)	0.99 ± 0.33	0.73 ± 0.08
ke (/h)	18.32 ± 1.74	8.31 ± 0.71

*AUC, area under the plasma concentration-time curve; CL, total body clearance; V_d_, volume of distribution; t_1/2_, elimination half-life time; k_e_, elimination rate constant.*

### Evaluation of the Analgesic Effect of Naringenin Solid Dispersion *via* the Acetic Acid Writhing Test

**FIGURE 5:**
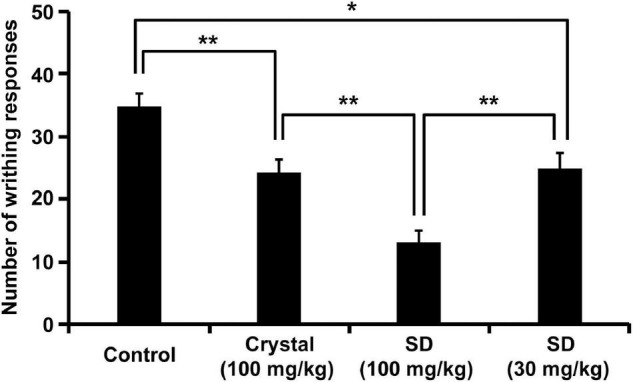
Inhibition of acetic acid-induced writhing behavior by NRG SD. Saline (control), crystalline NRG (100 mg/kg), and NRG SD (30 or 100 mg/kg) were orally administered five min before acetic acid treatment. Cumulative numbers of abdominal contortions (writhing score) were evaluated over 25 min (means ± SE; *n* = 8). *p < 0.05, **p < 0.01 (Tukey–Kramer method).

We conducted an *in vivo* functional evaluation of NRG SD to confirm its efficacy and assessed its analgesic effect because this property is a characteristic of NRG. To this end, we applied the acetic acid writhing test (8) by intraperitoneally administering acetic acid to the rats 5 min after oral administration of crystalline NRG and NRG SD and then enumerating the writhings after 25 min of administering acetic acid. Both the crystalline NRG and the SD treatments significantly decreased the number of writhings compared to the control (Figure 5). Rats administered SD equivalent to 30 mg/kg NRG showed the same degree of writhing suppression as those administered crystalline NRG but at one-third dose. This result suggests that SD demonstrated improved functionality than crystalline NRG.

### Naringenin Solid Dispersion Safety After 28 Days of Administration of Mixed Diet

To investigate the safety of NRG SD, we examined its *in vivo* effects when administered to mice for 28 days. Male and female mice were fed with a powdered diet including 1% (w/w) base materials (control) or 1% (w/w) NRG SD equivalent to 0.1% (w/w) NRG. There were no significant differences between the control and NRG SD groups or males and females in terms of body weight or water consumption (Supplementary Figure 1). Food intake increased significantly by day 9 for females administered SD and decreased by day 14 for males administered SD. Nevertheless, these changes were still within the normal range (28). The weights of the livers of the female mice administered NRG SD significantly increased relative to those of the control. However, there were no significant differences between different treatment groups or sexes in terms of the weights of any other organs (Table 3). We evaluated the hematological parameters of mice using blood samples collected after 28 days of feeding. There were no significant differences between the control and NRG SD groups in terms of these factors (Table 4). Biochemical and chemical serum parameters were then measured in mouse plasma to understand the effects of SD administration for 28 days on blood chemistry (Table 5). Relative to the control, the levels of alanine aminotransferase (ALT) were reduced significantly in male mice administered NRG SD. Nevertheless, the change was still within the normal range (29). There were no significant differences in any other clinical chemistry parameters associated with the liver, kidney, heart, or other organs between different treatment groups or sexes. The results suggest that NRG SD can be considered safe, and oral ingestion of this material should cause no adverse effects.

**TABLE 3:** Tissue weights (mg/g BW) of mice after administration of mixed diet including 1% (w/w) NRG SD or 1% (w/w) base materials (control) for 28 days.

Tissue (mg/g body weight)	Male		Female	
	Control	*SD*	Control	*SD*
Liver	54.35 ± 1.84	50.73 ± 1.39	43.95 ± 1.24	48.41 ± 0.68*
Spleen	3.40 ± 0.14	3.18 ± 0.11	4.10 ± 0.41	4.48 ± 0.15
Heart	4.71 ± 0.28	5.19 ± 0.24	5.14 ± 0.27	4.92 ± 0.30
Kidney	17.57 ± 0.23	18.30 ± 0.86	14.40 ± 0.78	14.21 ± 0.56
Lung	5.42 ± 0.16	5.48 ± 0.25	5.99 ± 0.43	6.10 ± 0.46
Brain	13.36 ± 0.47	12.81 ± 0.49	16.16 ± 0.93	15.04 ± 0.74

Statistical analyses were performed for a comparison between for control vs. SD for males and control vs. SD for females (mean ± SE; n = 5). *p < 0.05 (unpaired t-test).

**TABLE 4:** Hematological parameters of mice after administration of mixed diet including 1% (w/w) NRG SD or 1% (w/w) base materials (control) for 28 days.

Blood cells	Male		Female	
	Control	*SD*	Control	*SD*
White blood cell (× 10^2^/μL)	22.38 ± 3.64	13.60 ± 3.59	9.26 ± 2.46	21.10 ± 9.50
Neutrophil (× 10^2^/μL)	2.04 ± 0.37	1.92 ± 0.66	0.80 ± 0.20	1.52 ± 0.52
Lymphocyte (× 10^2^/μL)	18.10 ± 3.63	10.40 ± 2.33	8.28 ± 2.14	15.58 ± 7.49
Monocyte (× 10^2^/μL)	1.06 ± 0.24	0.86 ± 0.16	0.32 ± 0.17	1.14 ± 0.60
Eosinophil (× 10^2^/μL)	0.68 ± 0.08	0.48 ± 0.23	0.18 ± 0.07	1.46 ± 0.88
Red blood cell (× 10^4^/μL)	971.00 ± 23.85	961.60 ± 22.78	935.80 ± 20.89	922.60 ± 20.26
Hemoglobin (g/dL)	15.76 ± 0.43	15.60 ± 0.26	15.36 ± 0.20	15.38 ± 0.23
Hematocrit (%)	47.60 ± 1.10	47.26 ± 0.48	45.94 ± 0.62	46.12 ± 0.74

*Statistical analyses were performed for a comparison between control vs. SD for males and control vs. SD for females (means ± SE, n = 5, unpaired t-test method).*

**TABLE 5:** Test parameters of comprehensive clinical chemistry in mice after administration of mixed diet including 1% (w/w) NRG SD or 1% (w/w) base materials (control) for 28 days.

		Male		Female	
Analyte	Units	Control	*SD*	Control	*SD*
Albumin	g/dL	2.36 ± 0.05	2.32 ± 0.10	2.36 ± 0.08	2.33 ± 0.13
AST	U/L	79.40 ± 1.83	81.20 ± 4.82	117.20 ± 13.91	105.33 ± 17.27
ALT	U/L	39.00 ± 1.45	31.00 ± 1.22**	34.40 ± 3.34	43.00 ± 12.12
ALP	U/L	225.60 ± 4.24	241.80 ± 19.53	316.00 ± 43.29	256.33 ± 35.00
Total bilirubin	mg/dL	0.56 ± 0.07	0.60 ± 0.13	0.68 ± 0.04	0.70 ± 0.12
Direct bilirubin	mg/dL	0.18 ± 0.04	0.23 ± 0.06	0.22 ± 0.02	0.20 ± 0.06
LDH	U/L	832.00 ± 31.69	766.00 ± 49.18	936.00 ± 66.60	751.67 ± 28.92
GGT	U/L	ND	ND	ND	ND
LAP	U/L	61.40 ± 2.46	68.25 ± 7.59	58.60 ± 1.69	60.33 ± 3.84
Cholinesterase	U/L	12.20 ± 2.01	13.25 ± 2.17	30.00 ± 3.48	27.33 ± 4.33
Ammonia	μg/dL	173.80 ± 15.98	164.80 ± 21.11	180.60 ± 44.13	168.00 ± 24.01
CPK	U/L	638.80 ± 119.48	844.00 ± 151.91	711.00 ± 225.13	479.00 ± 80.00
CKMB	U/L	137.80 ± 8.63	145.25 ± 9.74	144.40 ± 6.47	142.00 ± 18.77
Amylase	U/L	3,384.00 ± 281.13	3,394.00 ± 165.65	3,031.00 ± 163.02	2,490.00 ± 395.38
BUN	mg/dL	31.28 ± 2.59	32.10 ± 3.63	30.24 ± 4.84	23.13 ± 1.62
Creatinine	mg/dL	<0.2	<0.2	<0.2	ND
Uric acid	mg/dL	1.80 ± 0.16	1.50 ± 0.12	1.66 ± 0.19	1.73 ± 0.22
Sodium	mEq/L	141.25 ± 0.85	142.75 ± 0.63	142.60 ± 1.21	142.33 ± 0.88
Potassium	mEq/L	7.68 ± 0.27	7.03 ± 0.42	7.46 ± 0.34	6.87 ± 0.35
Chloride	mEq/L	113.00 ± 1.08	112.25 ± 1.80	114.60 ± 0.81	113.67 ± 0.33
Magnesium	mg/dL	1.78 ± 0.04	1.86 ± 0.05	1.84 ± 0.09	1.87 ± 0.15
Calcium	mg/dL	9.54 ± 0.21	9.16 ± 0.07	9.72 ± 0.21	9.37 ± 0.23
Inorganic phosphorus	mg/dL	11.40 ± 0.51	9.94 ± 0.51	10.54 ± 0.68	11.83 ± 1.27
Total cholesterol	mg/dL	112.20 ± 5.77	116.00 ± 7.35	80.20 ± 8.16	80.00 ± 3.00
Triglycerides	mg/dL	121.60 ± 26.40	123.00 ± 36.41	89.40 ± 30.49	91.67 ± 2.85
Glucose	mg/dL	258.80 ± 12.99	219.20 ± 22.08	212.00 ± 7.71	219.00 ± 7.16

Statistical analyses were performed for comparison between control vs. SD for males and control vs. SD for females (mean ± SE; n = 5). **p < 0.01 (unpaired t-test).

*AST, aspartate aminotransferase; ALT, alanine aminotransferase; ALP, alkaline phosphatase; LDH, lactate dehydrogenase; GGT, γ-glutamyl transpeptidase; LAP, leucine aminopeptidase; CPK, creatine phosphorus kinase; CKMB, creatine kinase; MB isozyme; BUN, blood urea nitrogen; ND, not detected.*

## Discussion

SD technology increases the water solubility of certain compounds and their gastrointestinal absorption (30). Dispersing a compound in a biologically inert matrix, such as a polymer, reduces its molecular mobility and inhibits crystal reformation (31, 32). Based on this technology, we attempted to improve the dissolution profile of the compounds by inhibiting their nucleation and crystal formation *via* micellization with an emulsifier (15, 33). SDs prepared by thermal mixing of a mixture of poorly water-soluble β-carotene, a polymer, and an emulsifier dramatically improved the water solubility of β-carotene (15). In the present study, we used hot-melt extrusion technology to prepare NRG SD whose flavonoid skeleton structurally differed from the carotenoid skeleton of β-carotene. The solubility of NRG SD in water is ∼15 mg/mL, which was > 200-fold higher than that of crystalline NRG (Figure 1). In a formulation prepared by spray drying method using α-glycosyl hesperidin as a base material, the solubility of NRG was approximately 3.5 mg/mL (34), whereas that of NRG-β-cyclodextrin complex is ∼5 mg/mL (14). As the NRG SD prepared in this study had higher solubility than previously reported formulations, it was expected to demonstrate superior water solubility and gastrointestinal absorption compared to the previous NRG formulations. Additionally, the water solubility of NRG in PM (generated by mixing NRG, PVP, and PG, Figure 1) also increased. As the NRG hydroxyl group and the PVP carbonyl group form hydrogen interactions (35), the water solubility of NRG in PM was enhanced by the formation of soluble complexes. However, NRG SD is considered more efficacious because the water solubility of NRG in NRG SD is more than threefold higher than that of NRG in PM.

The dissolution tests demonstrated that 100% NRG SD dissolved within 20–30 min. In contrast, crystalline NRG dissolution was considerably slower (Figures 1B,C). It was found that 100% of the NRG-loaded nanoparticles dissolved in simulated gastric fluid within ∼40 min (10). The NRG formulation prepared *via* the self-nanoemulsifying drug delivery system required ∼45 min for 100% dissolution in phosphate buffer (pH 6.8) (12). Our NRG SD dissolved faster than the foregoing NRG formulations and could, therefore, achieve superior gastrointestinal absorption. The amorphization of NRG was a fundamental factor enhancing its solubility as observed in the present study. The NRG SD diffraction peaks were examined using PXRD analysis. The disappearance of the crystalline NRG peaks suggested that NRG SD was in an amorphous state (Figure 2B). In contrast, no crystalline NRG peaks were detected in the DSC analyses of NRG SD and PM. Thus, the amorphous state of NRG SD could not be established using this analytical method (Figure 2A). It is unknown why the crystalline NRG peak disappeared in PM. Gera et al. reported a similar phenomenon for the DSC analysis of NRG nanosuspensions (36). The authors used PVP-K90 as the polymer in their formulation. PVP is known to interact with NRG (35). The preceding findings suggest that the interaction between NRG and PVP may have altered the crystalline state of NRG in PM. Hence, we believe that the crystalline NRG peak may have disappeared.

Flavonoids undergo sulfation, methylation, and glucuronidation in the small intestine and the liver (37). Most aglycones do not appear in the plasma. NRG glucuronide and sulfate conjugates have previously been reported (38). Flavonoid metabolites are usually less bioactive than their parent compounds. Nevertheless, reports have been published indicating the contrary (39). In the present study, we measured the amounts of unchanged NRG and NRG glucuronate and sulfate conjugates in the plasma to determine NRG SD absorption in the gut after oral administration (Figure 3). For NRG SD, C_max_ and AUC_0–1 h_ increased and t_max_ decreased regardless of the NRG status because NRG SD rapidly dissolved in the test solutions (Figure 1). Orally administered NRG SD might have dissolved quickly in the rat gastrointestinal tract. Hence, a high concentration of NRG was absorbed in the gut and maintained thereafter. Relative to unchanged NRG, the concentration of NRG SD was higher than those of crystalline NRG and NRG PM. By contrast, AUC_0–∞_ did not markedly differ among crystalline NRG, NRG PM, or NRG SD metabolites (Table 1). Figure 1 shows that the dissolution behaviors of crystalline NRG and NRG PM approached that of NRG SD over time. Thus, all three formulations will eventually yield similar NRG concentrations upon dissolution in the gastrointestinal tract. For this reason, it was difficult to detect any differences in AUC_0–∞_ among the formulations.

We intravenously administered physiological serum containing unchanged NRG and NRG metabolites to evaluate true pharmacokinetic parameters of NRG (Figure 4 and Table 2). Prior pharmacokinetic analyses involved intravenous administration of NRG dissolved in organic solvents or polymers because NRG has poor water solubility (40, 41). The effects of injected organic solvents and polymers cannot be eliminated when a dosing solution is used. Hence, it is uncertain whether the results obtained in those assays reflect the actual trends observed in blood NRG concentrations under physiological conditions. In the present study, we prepared serum samples with the highest NRG concentration obtained after oral NRG SD administration and used it in the intravenous administration test. This experiment was feasible because our SD had high C_max_ (18). The pharmacokinetic analysis indicated that the distribution volume of the unchanged NRG and NRG metabolites was equivalent to ∼20% of the body weight and that the elimination rate constant k_e_ was high (Table 2). NRG has a high affinity for albumin; thus, only a small quantity of NRG would be transferred from the blood to the tissue (42). These findings suggest that NRG shows low tissue migration and rapid excretion rates. The absolute bioavailability of NRG SD was ∼2.7-fold greater than that of crystalline NRG (Table 1), whereas the absolute bioavailability of orally administered crystalline NRG was only ∼0.5% in rats. Relative to the oral NRG dose, the cumulative urinary excretion rate in humans was reported to be ∼5.8% (43), which is one order of magnitude higher than that in rats. Bai et al. reported pharmacokinetic differences among humans, dogs, and rats in terms of NRG disposition. The difference among species was also considered an important factor in the present study (38).

In this study, we explored the analgesic effect of NRG as one model to demonstrate the efficacy of NRG SD. In the acetic acid writhing test, NRG SD demonstrated stronger analgesic effects than crystalline NRG (Figure 5). Treatment with 30 mg/kg of SD provided one-third the dose of NRG available with 100 mg/kg of crystalline NRG treatment; however, both treatments had comparable analgesic efficacy. This finding suggests that NRG SD is an excellent formulation with high pharmacological efficacy even at low doses. Our acetic acid writhing test evaluated the analgesic effect of external chemical stimulation against nociceptive pain. NRG also exhibited analgesic activity in the tail-flick method, which involves eliciting pain by thermal stimulation. Thus, it is expected that NRG SD demonstrates analgesic efficacy against several painful conditions (44). The primary mechanism of NRG-based analgesia involves the inhibition of NF-κB activation and by extension, that of inflammatory cytokine production. Moreover, NRG-based analgesia does not demonstrate side effects associated with non-steroidal anti-inflammatory drugs (NSAIDs), such as the increased risk of gastrointestinal disorders, and may therefore help improve the quality of life of patients (8, 45). Hence, NRG SD can be an effective functional food for pain relief. Furthermore, it is possible that NRG SD can be widely used based on the antioxidant, anti-inflammatory, and anti-obesity effects of NRG in addition to its analgesic effects.

The safety and quality of NRG SD as a functional food should also be established for its application in clinical practice. The water solubility of NRG SD stored at room temperature for 2 years was 15.3 ± 0.1 mg/mL, which was the same as that of the original preparation, demonstrating the stability of the product. PXRD analysis also showed that the solution was not crystallized during that time (data not shown). Thus, NRG SD can remain stable even after long-term storage. NRG SD safety was evaluated after administration to mice for 28 days (Tables 3–5 and Supplementary Figure 1). However, there was a significant increase in the liver weight of female mice orally administered NRG SD (Table 3). Since NRG is a ligand for the nuclear receptor peroxisome proliferator-activated receptor (PPAR)α (46), which induces liver hypertrophy and hyperplasia in rodents (47), the observed increase in mouse liver weight can be explained by liver hypertrophy and hyperplasia induced by the binding of NRG to PPARα. Nevertheless, PPARα ligand-induced hepatomegaly is not observed in humans. PPARα ligands are clinically administered for the treatment of hyperlipidemia (48). Moreover, liver function parameters such as albumin, aspartate aminotransferase (AST), ALT, alkaline phosphatase (ALP), total bilirubin, direct bilirubin, lactate dehydrogenase (LDH), γ-glutamyl transpeptidase (GGT), leucine aminopeptidase (LAP), cholinesterase, and ammonia demonstrated normal values in the NRG SD-treated animals in the present study (Table 5). Therefore, NRG SD can be considered a safe functional food. The results of this study suggest that the routine use of NRG SD has the potential to effectively and economically maintain human health and reduce the risk of chronic diseases.

## Conclusion

In this study, the water solubility and gastrointestinal absorption of the NRG SD prepared were superior to those of crystalline NRG. Furthermore, NRG SD displayed high analgesic activity and did not demonstrate any major adverse effects in mice that were administered with NRG SD for 28 days. Our hot-melt extrusion technology can be successfully used to prepare amorphous solid dispersions and was previously applied to β-carotene, which has a carotenoid skeleton. This study demonstrated that hot-melt extrusion technology could also be applied to substances such as NRG with polyphenol flavonoid skeletons. Hence, our methodology has the potential to improve the water solubility of a wide range of compounds with diverse structures. In the future, we will validate the safety and efficacy of NRG SD in human clinical trials and develop practical disease prevention and treatment protocols using NRG SD as a functional food material.

## Data Availability Statement

The original contributions presented in the study are included in the article/Supplementary Material, further inquiries can be directed to the corresponding author/s.

## Ethics Statement

The animal study was reviewed and approved by the Animal Care and Use Committee of the Graduate School of Pharmaceutical Sciences Osaka University, Osaka, Japan.

## Author Contributions

KI designed the study and drafted the manuscript. YS, AO, SM, BL, and KN collected the test data. SO, YA, NH, and MS helped in the interpretation of results. SN directed the research and reviewed the manuscript. All authors contributed to the article and approved the submitted version.

## Conflict of Interest

SO, SM, and MS were employed by Mitsui Norin Co., Ltd. The remaining authors declare that the research was conducted in the absence of any commercial or financial relationships that could be construed as a potential conflict of interest.

## Publisher’s Note

All claims expressed in this article are solely those of the authors and do not necessarily represent those of their affiliated organizations, or those of the publisher, the editors and the reviewers. Any product that may be evaluated in this article, or claim that may be made by its manufacturer, is not guaranteed or endorsed by the publisher.
