# Supplementary material for: Physicochemical and Biochemical Evaluation of Amorphous Solid Dispersion of Naringenin Prepared Using Hot-Melt Extrusion
Source: Front Nutr. 2022 Apr 27;9:850103. doi: 10.3389/fnut.2022.850103 (PMC9093646; doi:10.3389/fnut.2022.850103)
Supplement: Supplementary file 1 [file Image_1.pdf]

Supplementary Material

1 Supplementary Tables and Figures

1.1 Supplementary Figures

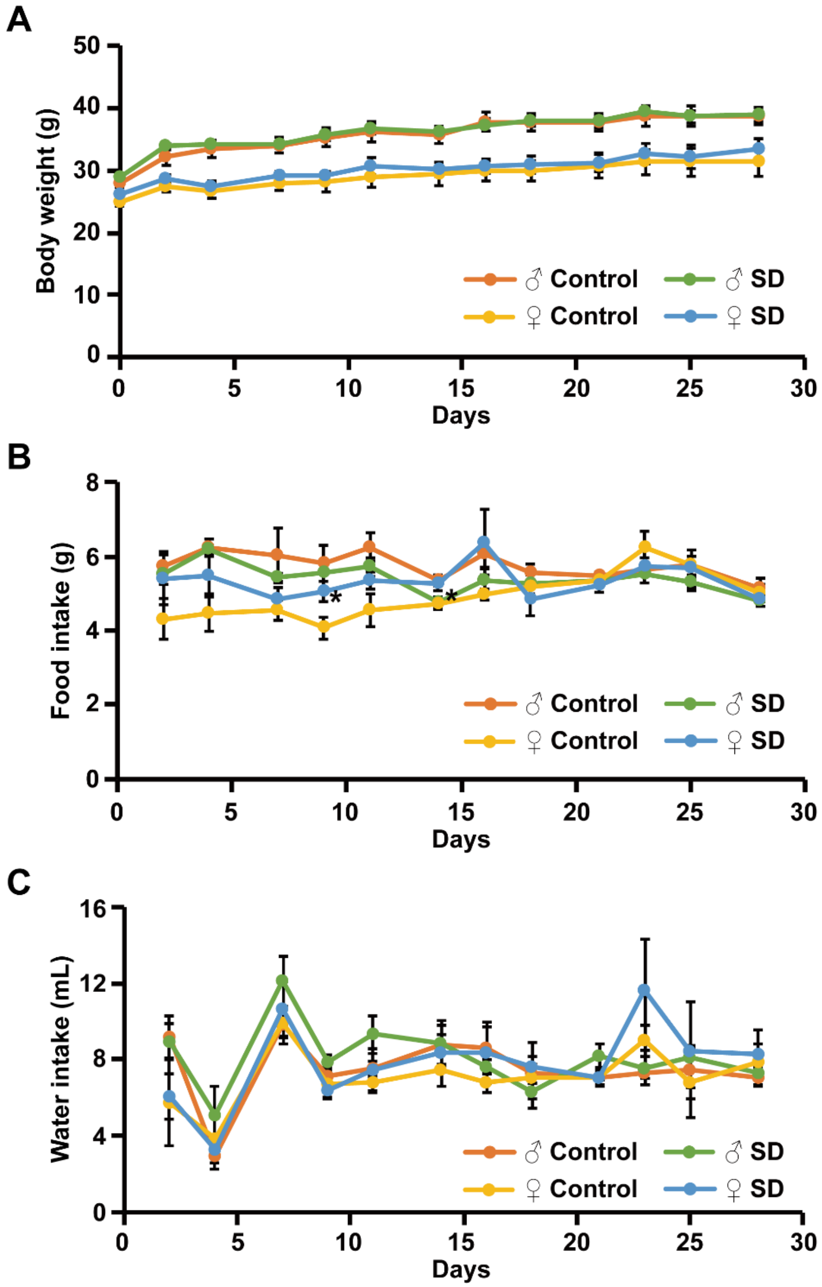

Supplementary Figure 1. Changes in body weight (A), food intake (B), and water intake (C) after administration of mixed diet containing 1% (w/w) NRG SD or 1% (w/w) base materials (control) to mice for 28 d. Statistical analyses were performed for comparing control vs. SD for males and control vs. SD for females (mean ± SE; n = 5). *$p < 0.05$ (unpaired *t*-test).
